# Absence of increased genomic variants in the cyanobacterium *Chroococcidiopsis* exposed to Mars-like conditions outside the space station

**DOI:** 10.1038/s41598-022-12631-5

**Published:** 2022-05-19

**Authors:** Alessandro Napoli, Diego Micheletti, Massimo Pindo, Simone Larger, Alessandro Cestaro, Jean-Pierre de Vera, Daniela Billi

**Affiliations:** 1 Department of Biology, University of Rome Tor Vergata, Via della Ricerca Scientifica snc, 00133 Rome, Italy; 2 PhD Program in Cellular and Molecular Biology, Department of Biology, University of Rome Tor Vergata, Rome, Italy; 3 Edmund Mach Foundation, via E. Mach 1, 38010 San Michele all’Adige, Italy; 4 German Aerospace Center (DLR), Microgravity User Support Center, Linder Höhe, 51147 Cologne, Germany

**Keywords:** Astrobiology, Genome informatics

## Abstract

Despite the increasing interest in using microbial-based technologies to support human space exploration, many unknowns remain not only on bioprocesses but also on microbial survivability and genetic stability under non-Earth conditions. Here the desert cyanobacterium *Chroococcidiopsis* sp. CCMEE 029 was investigated for robustness of the repair capability of DNA lesions accumulated under Mars-like conditions (UV radiation and atmosphere) simulated in low Earth orbit using the EXPOSE-R2 facility installed outside the International Space Station. Genomic alterations were determined in a space-derivate of *Chroococcidiopsis* sp. CCMEE 029 obtained upon reactivation on Earth of the space-exposed cells. Comparative analysis of whole-genome sequences showed no increased variant numbers in the space-derivate compared to triplicates of the reference strain maintained on the ground. This result advanced cyanobacteria-based technologies to support human space exploration.

## Introduction

As humans will once again travel to the Moon and eventually settle on Mars, autonomy from Earth is mandatory since consumable re-supply is challenging due to launch costs, travel times and failure risks. Hence there is an increasing interest in advancing life support systems including bioregenerative life support systems to provide oxygen and food^1^ and biotechnology/synthetic biology applications for on-demand production^2,3^. However, many unknowns remain when moving microbial-based technologies outside the laboratory-settings on Earth. Main challenges consist in a reduced or absent gravity combined with space radiation effecting not only microbial bioprocesses but also genetic stability over prolonged periods and variable storage conditions^2^. Technologies known as in-situ resource utilization (ISRU) hold promises in enhancing long-term self-sustaining human outposts on the Moon or on Mars by conferring the capability to live off the land^3^. Cyanobacteria-based technologies are relevant since might take advance of their capability of producing oxygen and fixed carbon, using local resources, namely carbon dioxide from the Martian atmosphere and nutrients from lunar and Martian soil^4^.

The knowledge gathered over 50 years of astrobiology experiments largely contributed to unravel the effects of the space conditions on terrestrial microorganisms. These experiments aim to address two main scientific questions: What does microbial resistance to space tell us about life beyond Earth? What can we learn from space experiments to support future exploration missions?^5^. Indeed, the investigation of microbial resilience in space started with human space exploration. First experiments demonstrated that bacterial spores could survive space conditions when mounted on sounding rockets as well as deep-space exposure during the Apollo 16’s return to Earth^5,6^. Later, bacterial spores were reporter to survive 1- and 6-year space exposure using the NASA LDEF (Long Duration Exposure Facility) and the ESA EURECA (EUropean REtrievable Carrier) free-flying, respectively^5,6^. Starting from the late 2000s the ESA EXPOSE facility installed outside the International Space Station (ISS) allowed long-term space exposures (about 2 years) of a wide variety of desiccation-, radiation-tolerant microorganisms^5^. Since microorganisms were exposed in the dried state to Mars-like conditions, their re-activation after return to Earth suggested the repair of the accumulated DNA damage^5^.

The potential of microbial-based technologies beyond Earth was demonstrated by the ESA BioRock experiment performed on-board the ISS^7,8^. After transport through space in the dried state, desiccation-tolerant bacteria were re-activated under Mars-simulated gravity and showed unaltered bioleaching activities from basalt, a material found on the Moon and Mars^7,8^. While bacteria used in BLSS could be re-activated on Earth after 7-day storage in the hydrated state on-board the ISS, exception made for the edible cyanobacterium *Arthrospira* sp. PCC 8005^9^.

Astrobiology experiments performed in low Earth orbit (LEO) using the ESA EXPOSE facility contribute to pioneering cyanobacteria-based life support technologies beyond Earth^10^. Cyanobacteria of the genus *Chroococcidiopsis* isolated from Mars-like deserts, being suitable to genetic manipulation hold promises for space biotechnology/synthetic biology applications^11–13^. Furthermore, being lithotrophic they could grow using Moon or Mars regolith as nutrient source, and hence provide a link between ISRU and already developed life support systems^7,10^. For instance, by producing organic compounds, cyanobacteria could feed bacteria used as chassis in biotechnology/synthetic biology^14^. As a proof-of-concept a cyanobacterial lysate was used to grow a *Bacillus subtilis* strain engineered to synthetize aromatic polymers proposed for space applications^15^. In addition, during the NASA space experiment PowerCell, a cyanobacterial lysate was used to germinate *Bacillus subtilis* spores^16^.

Evidence for the capability of the desert cyanobacterium *Chroococcidiopsis* sp. CCMEE 029 of repairing DNA damage accumulated while in space were obtained during the astrobiology experiments performed using the ESA EXPOSE-R2 facility outside the ISS^17,18^. Post-flight analysis performed in the context of the BIOlogy and Mars EXperiment (BIOMEX) space experiment showed that dried *Chroococcidiopsis* cells mixed with Martian regolith survived 1.5-year exposure to space radiation and a Mars-like environment simulated in LEO. This feature supported the use of this cyanobacterium for space technologies, although it is first mandatory to assess whether upon re-activation there is a robust repair of the accumulated DNA damage.

Here the desert cyanobacterium *Chroococcidiopsis* sp. CCMEE 029 was investigated for robustness of the repair of DNA lesions accumulated under Mars-like conditions simulated in LEO. Therefore, genomic alterations were investigated in a space-derivate strain obtained upon rehydration of dried cells exposed for 1.5 years to cosmic ionizing radiation and Mars-like conditions (UV radiation and atmosphere) simulated outside the ISS during BIOMEX space experiment^19^. Comparative analysis of the whole-genome sequences obtained using Illumina MiSeq and Oxford Nanopore MinION platforms, showed no increased variants in the space-derivate compared to independent triplicates of the reference strain maintained on the ground.

## Results

### Genome assembly of space-derived and ground-reference strains

**Figure 1:**
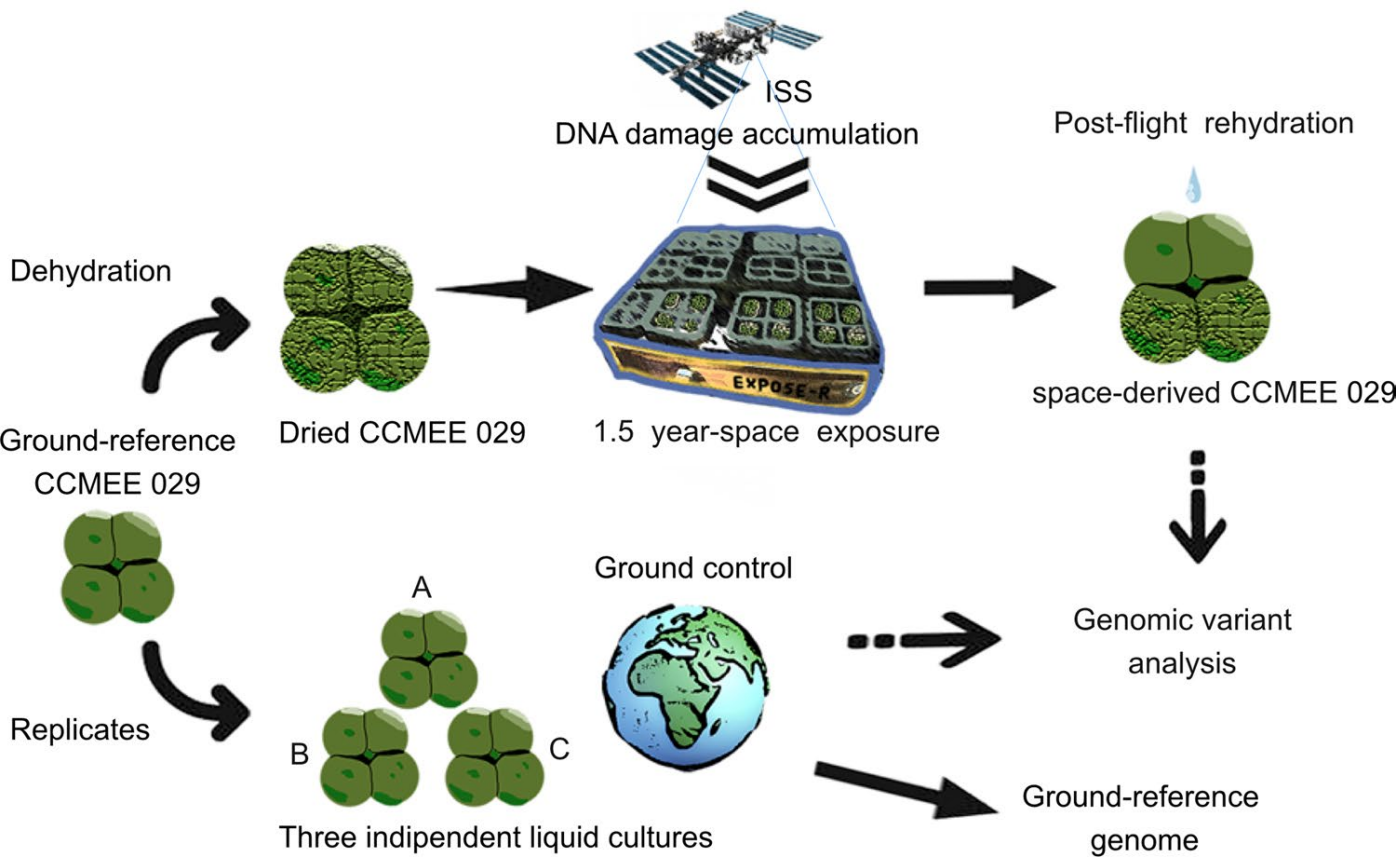
Experimental design. During the BIOMEX space experiment, dried cells of *Chroococcidiopsis* sp. CCMEE 029 were exposed to cosmic ionizing radiation combined with a Mars-like environment (UV radiation and atmosphere) simulated in LEO by using the ESA EXPOSE-R2 facility installed outside the ISS. The space-derivate was obtained upon rehydration of the exposed sample after retrieval to Earth. Whole-genome comparative analyses were performed among liquid triplicates of the ground-reference strain and the space-derived strain.

During the BIOMEX space experiment a dried sample of the cyanobacterium *Chroococcidiopsis* sp. CCMEE 029, mixed with Martian regolith simulant, was exposed for 1.5-years to cosmic ionizing radiation combined with a Mars-like environment (UV radiation and atmosphere) simulated by using the EXPOSE-R2 facility (Fig. 1). After return to Earth, a space-derived strain was obtained upon rehydration of the exposed sample, while the ground-reference strain was maintained in independent triplicates (Fig. 1). The genomes of the ground-reference triplicates and of space-derivate were successfully assembled by combining Illumina and Oxford Nanopore sequencing technology (ONT) reads.

Paired-end DNA libraries yielded 1.76, 2.06 and 1.24 millions of reads for the ground-triplicates, CCMEE 029 A, B and C, respectively, and 3.9 millions of reads for the space-derived strain, while 500.4, 96.7, 471.8 and 135.5 thousands ONT reads were obtained for the ground-triplicates CCMEE 029 A, B and C and space-derived strain, respectively (Supplementary Table 1). A ground-reference genome was obtained from the reads generated from the libraries of ground-reference triplicates. Before filtering for non-cyanobacterial contigs, the ground-reference assembly resulted in 115 contigs with the longest contig of ~ 5.7 Mbp, while the space-derived assembly in 75 contigs with the longest contig of ~ 4.9 Mbp (Supplementary Table 2). The taxonomy classification of the contigs and the size distribution of their taxonomy classes were assigned (Supplementary Tables 3 and 4; Supplementary Fig. 1). Non-cyanobacterial sequences were revealed by the GC-content distribution and k-mer spectra analysis (Supplementary Figs. 2 and 3) and their presence ascribed to bacteria present in *Chroococcidiopsis* cell envelope^20^.

The Kraken's analysis showed that the genome with the highest similarity to the longer CCMEE 029 contig corresponded to the cyanobacterium *Nostoc punctiforme* PCC 73102. Additional contigs greater than 1 Mbp were classified as *Zymomonas mobilis* subsp. *mobilis* NCIMB 11163 (3.8 Mbp) and *Brevundimonas subvibrioides* ATCC 15264 (1.2 Mbp). After the Kraken analysis of the ground-reference assembly, non-cyanobacterial contigs were filtered out and two contigs remained: a single circular contig, likely a 673-Kbp plasmid and a ~ 5.7-Mbp chromosome (Supplementary Table 3). The contigs of the space-derived assembly were 11 with a total length of ~ 6.3 Mbp (Supplementary Table 4).

### Genome annotation

A total of 6388 genes were identified in the ground-reference genome and the species distribution was assessed (Supplementary Fig. 4). The space-derived reads were mapped on the ground-reference genome, and 23.3% of paired-end reads and 44.5% of long reads were aligned. The aligned reads covered almost the whole ground-reference genome with an average depth of 382 and 481 for short and long reads, respectively.

The functional annotation yielded a prediction for 3549 out of 6388 genes, BlastKOALA provided KEGG terms for 4018 genes and Blast2GO returned an annotation for 4095 genes; in particular, 3212 genes were associated to molecular function terms, 2657 to biological processes terms and 1634 to cellular components terms. Based on the most representative classes of Gene Ontology annotation the majority of the cellular component terms were referred to the category integral component of membrane (63%), the highest percentage of biological process belonging to cellular macromolecule biosynthetic (14%), followed by phosphate-containing compound metabolic process (12%), DNA metabolic process (11%), oxidation–reduction process (10%) and regulation of cellular process (10%), while the other biological process were retrieved in lower percentage (Supplementary Fig. 5). Among the molecular functions, the transferase activity (32%) was the most abundant, followed by hydrolase activity (24%), oxidoreductase activity (15%), metal ion binding (11%), DNA binding (10%) and ATP binding (8%) (Supplementary Fig. 5).

### Comparative analysis and annotation of single nucleotide variants (SNVs) and insertion or deletion mutation (InDels)

**Figure 2:**
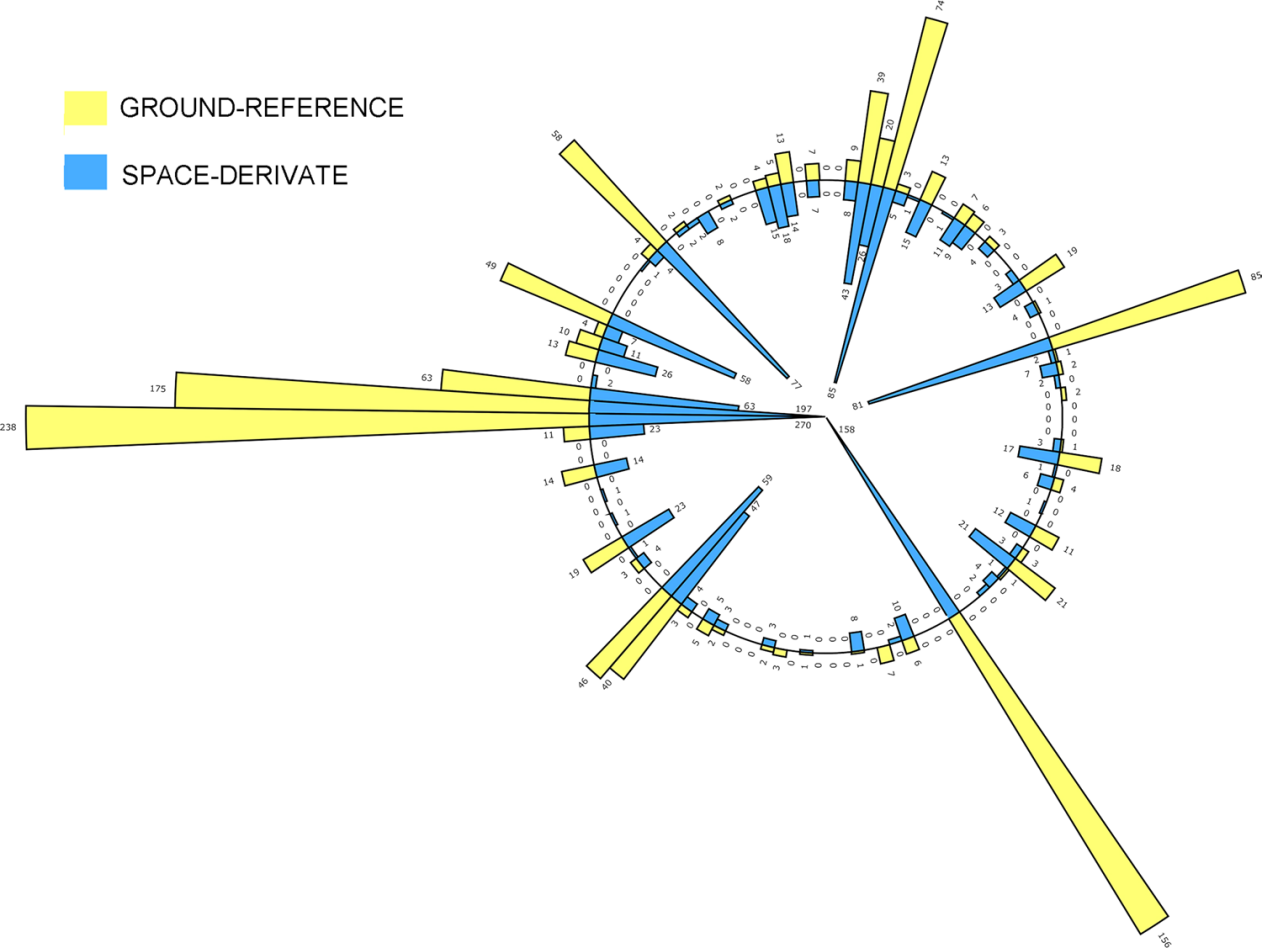
Circular bar plots showing of single nucleotide variants (SNVs) in the ground-reference *Chroococcidiopsis* sp. CCMEE 029 and space-derivate. The yellow bar plot shows the SNV frequency in the ground-reference genome representing the sum, according to the set theory, of the SNVs of each ground-reference replicate (CCMEE 029 A, B and C). The light-blue bar plot shows SNVs frequency in the space-derived genome. Bars indicate the SNVs frequency in 50-kbp sectors, numbers show absolute frequency values.

The SNV and InDels were identified by comparative analysis of Illumina reads of the ground-reference triplicates and space-derivate against the ground-reference genome. The total number of SNVs and InDels found in the ground-reference triplicates (CCMEE 029 A, B and C) and in the space-derivate was 1857, 1582, 1046 and 1603, respectively. Variants with an identical genotype in each one of the four samples were removed as considered not related to the space exposure, and the remaining variants were 1645, 1370, 836 and 1392 for the ground-reference triplicates and for the space-derived strain, respectively. An average nucleotide diversity of 1 variant every 4.8 kbp resulted for the three ground-reference replicates and 1 variant every 4.1 kbp for the space-derived strain (Table 1). An average of 16 insertions and 18 deletions were present in the ground-reference triplicates (CCMEE 029 A, B and C), while 15 insertions and 17 deletions were identified in the space-derived sample (Table 1). The most common variants were SNVs with a value of 1309 for the ground-reference genome obtained by summing the SNVs of each ground-reference replicate, and of 1360 for the space-derived genome (Fig. 2). In addition, a comparable presence of hot spots for SNVs was evident in 50-kbp sectors of the ground-reference genome and space-derivate (Fig. 2).

**Table 1:** Variant detection in the genome of ground-reference triplicates of the ground-reference of *Chroococcidiopsis* sp. CCMEE 029 and space-derivate.

	Ground-reference			Space-derived
	A	B	C	
N° of variants	1645	1370	836	1392
Variants rate	3475	4172	6837	4106
SNV	1600	1335	814	1360
INS	21	18	9	15
DEL	24	17	13	17

*SNV* single nucleotide variant, *INS* insertion, *DEL* deletion.

The functional annotation of the ground-reference genome allowed the identification of genes affected by variants due to mutation position. The number of genes with variants were 309, 206 and 177 for the ground-reference triplicates, CCMEE 029 A, B and C, respectively, and 277 for the space-derived strain. Ground-reference triplicates and space-derived sample shared 124 affected genes (Supplementary Table 5), while ground-reference triplicates shared 233 genes with at least one variant, and 103 genes with at least one variant occurred only in the space-derivate.

The functional categories of the 124 affected genes shared by ground-reference triplicates and space-derived sample were identified (Supplementary Fig. 6A). The 233 affected genes of the ground-replicates and the 103 genes of the space-derived strain belong to the same functional categories (Supplementary Fig. 6B,C).

The percentage of synonymous variants, calculated on the variants within the coding region, were 59%, 62%, 64% and 63% for CCMEE 029 A, B, C and space-derivate, respectively, while the non-synonymous variants were the 40%, 37%, 35% and 36%. Furthermore, 8 stop-gain variants were found in the space-derivate and an average of 9 in the ground-reference triplicates. The stop-codons gained with shared KEGG annotation in the ground-reference triplicates and space-derivate, were two IS6 family transposase (GO:0015074) and one GNAT family *N*-acetyltransferase (GO:0008080). Ground-reference triplicates shared one RidA family protein (IPR035959 (G3DSA:3.30.1330.GENE3D); IPR035959 (SUPERFAMILY), and one SRPBCC domain-containing protein (IPR023393 (G3DSA:3.30.530.GENE3D); IPR013538 (PFAM); PTHR36929 (PANTHER); PTHR36929:SF4 (PANTHER); SSF55961 (SUPERFAMILY)). Finally, one IS630 family transposase (PF13518 (PFAM); IPR025959 (PFAM); IPR038717 (PFAM); G3DSA:3.30.420.470 (GENE3D); PTHR23022 (PANTHER); IPR009057 (SUPERFAMILY) and one WecB/TagA/CpsF family glycosyltransferase (P:GO:0009058; F:GO:0047244) were present only in the space-derivate (Supplementary Table 6).

### Structural variants (SVs)

SVs were identified by comparative analysis of the Illumina and Nanopore reads of the ground-reference triplicates and space-derivate against the ground-reference genome**.** The SVs identified in the ground-reference triplicates (CCMEE 029 A, B and C) and in the space-derivate were respectively 30, 11, 5 and 10. The SVs sharing the same genotype in all four samples were removed as well as the SVs longer than 10 kbp because lacking a strong support after alignment visual inspection. In the ground-reference replicate CCMEE 029-A, 10 variants passed the filters, corresponding to 4 deletions, 2 insertions and 4 duplications. The 4 variants retained in the ground-reference replicate CCMEE 029-B were one deletion, two deletions and one duplication. In the ground-reference replicate CCMEE 029-C, all the variants were filtered out. In the space-derived sample 3 variants were kept, one deletion, one insertion and one duplication. All the SVs found in the space-derived strain occurred in at least one of the ground-reference replicates CCMEE 029-A and 029-B and therefore considered as background noise.

Finally, the alignment performed with the MUMmer package of the longest-assembled scaffold of the space-derived sample (size 4,897,168 bp) with the ground-reference genome, yielded 7 unique and 748 repetitive alignments. The 7 alignments covered the whole ground-reference genome and were contiguous, all the alignments were collinear and there was no evidence for inversions, duplications or translocations.

## Discussion

Here the repair robustness of DNA lesions accumulated under Mars-like conditions simulated in low Earth orbit, was demonstrated for the desert cyanobacterium *Chroococcidiopsis* CCMEE 029, thus providing a first step towards its employment in life support technologies for space settlements. Indeed, during the BIOMEX space experiment DNA damage was accumulated in dried cells of this cyanobacterium exposed for 1.5 years to cosmic radiation and Mars-like simulated by using the ESA EXPOSE-R2 facility installed outside the ISS conditions^18,19^. Therefore, the space-derivate obtained upon rehydration of the exposed sample after retrieval to Earth^19^, was investigated for the presence of genomic alterations caused by an erroneous repair of the accumulated DNA lesions. The comparison of the whole-genome sequences of the space-derivate and independent triplicates of the ground-reference strain, revealed no increased variants in the space-derivate. Indeed, the nucleotide diversity of the space-derivate was comparable to that of the ground-reference triplicates. All the variants (single nucleotide variants, insertion and/or deletions) found in the space-derivate occurred in at least one of the ground-reference triplicates. The alignment between the ground-reference-genome and the longest-assembled scaffold of the space-derivate showed the absence of structural rearrangements. In the space-derivate the number of variants potentially affecting protein function, like stop codons or missense mutations, was comparable to that of each ground-reference replicate. As observed for each stop-codon gain found in the ground-reference replicates, the two predicted stop-codon gains occurring in the space-derivate, did not involve any known proteins of DNA repair pathways.

The absence of increased genomic variants in the space-derivate of C*hroococcidiopsis* sp. CCMEE 029 suggested no failure in the repair of DNA lesions caused by cosmic ionizing radiation and Mars-like UV flux simulated during the EXPOSE-R2 space mission^19^. This result is relevant when compared to the 4-order magnitude increase of rifampicin resistance and sporulation deficiency reported for *B. subtilis* spores exposed for 1.5 years to Mars-like UV flux during the EXPOSE-E space mission^25^. In addition, the genome stability of the space-derivate of C*hroococcidiopsis* sp. CCMEE 029 was not surprising considering its capability of withstanding DNA-damaging conditions, like 4 years of air-dried storage^21^, 1.5 years of space vacuum^22^, and 24 kGy of γ-radiation^23^. Moreover, as dried monolayers this strain survived 30 kJ/m^2^ of a Mars-like UV flux, thus resulting 10 times more resistant than *B. subtilis* spores^24^.

The robustness of the DNA repair taking place upon the reactivation of the space-exposed C*hroococcidiopsis* CCMEE 029 provided a significant step-change in its employment in biotechnology/synthetic biology applications to support human space exploration. In fact, during the BIOMEX space experiment dried CCMEE 029 cells received a dose of about 0.5 kGy of ionizing radiation and were re-activated after 900 days of desiccation^19^. Therefore, it might be anticipated that dried samples of this cyanobacterium can be transported to Mars and rehydrated after months of dry storage. Moreover, since the NASA Curiosity rover measured an ionizing-radiation dose of 76 mGy/year at Gale Crater^26^, at no point the Martian ionizing radiation environment would be lethal to this cyanobacterium. Moreover, during the BIOMEX space experiment, dried CCMEE 029 cells, mixed with a Martian regolith simulant, were exposed to a total dose of 2.19 × 10^2^ kJ/m^2^ of UV radiation (200–400 nm), corresponding to about 4 h-exposure on the Martian surface^18^. Indeed, a possible scenario of exposure to low-pressure and full UV-irradiation must be taken into account when foreseeing microbial-based technologies for space settlements^3^. The robustness of repair of the DNA lesions induced by a Mars-like UV flux is relevant when developping *Chroococcidiopsis*-based ISRU technologies^28^ aimed, for instance, to provide fixed carbon to heterotrophs commonly used in BLSS. As a proof-of-concept both the ground-reference C*hroococcidiopsis* sp. CCMEE 029 strain and its space-derivate tolerated a Mars-relevant perchlorate concentration, i.e., 2.4 mM perchlorate ions, as reported by the Phenix rover^29^, and their lysate could be used to support the growth of the bacterium *Escherichia coli*^28^.

No doubt desiccation-, and radiation-tolerant microorganisms, like *Chroococcidiopsis* sp. CCMEE 029, are advantageous when facing the challenges of moving technologies from the laboratory-settings beyond Earth^3,10^. Despite the last advances in cyanobacterial biotechnology/synthetic biology, additional improvements are required to make them competitive with bacteria-based technologies^30^. However, the availability of *Chroococcidiopsis* sp. CCMEE 029’s whole-genome sequence and the evidence for the robustness of the repair of DNA lesions accumulated under space radiation and Mars-like UV flux, provided a pre-requisite to investigate how microorganisms respond to non-Earth conditions. The EXPOSE facility allows the exposure of dried cells in which DNA damage is caused by the direct interaction between radiation and biological matter, while indirect damage induced by radical production is avoided^6^. Therefore, space experiments on metabolically active microorganisms are foreseen^5^ in order to unravel how the prolonged exposure to lunar or Martian conditions influences genome stability over multiple generation. Such an endeavor will take advantage of omics-based technologies that are rapidly developing for applications beyond LEO and currently tested on-board the ISS^31–33^. Notably, an automated, miniaturized gene expression device was validated using the cyanobacterium *Synechococcus elongatus,* thus offering a high throughput instrument for future deployment in space platforms other than ISS^34^.

## Methods

### BIOMEX space experiment

BIOMEX was a space experiment selected by the European Space Agency in response to the International Life Science Research Announcement (ILSRA-2009) for research in space life sciences at the ISS, by using the EXPOSE-R2 facility^35^. The BIOMEX experiment began on July 24, 2014 with the launch to the ISS of the Russian cargo ship progress 56, the exposure of the EXPOSE-R2 facility started on August 23, 2014 until samples retrieval inside the ISS on February 3, 2016 and final return back to Earth on June 18, 2016 with the Soyuz TMA-19M spacecraft^19^. During the BIOMEX space experiment, dried cells of *Chroococcidiopsis* sp. CCMEE 029 were mixed with the Martian simulant P-MRS and allocated in the 2-1-t-12 position of the EXPOSE-R2 facility (Fig. 1 in Ref.^19^). Due to the limited number of positions available in the EXPOSE-R2 facility^5^ only one sample of dried *Chroococcidiopsis* cells mixed with P-MRS was integrated^19^. The reduced sample amount left after post-flight assessment of cellular viability and DNA damage of the cells mixed with sulfatic Martian regolith simulant (S-MRS) impaired further investigations^19^. The mission environmental parameters were previously described^35^. The mission environmental parameters were described^35^. The cosmic radiation profile was measured with the Radiation Risks Radiometer–Dosimeter installed on the EXPOSE-R2 facility^36^, while the UV-fluence values were calculated by RedShift taking according to the sample position in the hardware and the shadowing due to the ISS orbit^18^. Temperature values below – 20 °C were prevented by the associated heating system prevented while the highest temperature measured during the mission was 57.98^35^. The BIOMEX sample here investigated was exposed to Mars-like UV flux (2.19 × 10^2^ kJ/m^2^ of UV 200–400 nm; 30 kJ/m^2^ UV 254 nm) combined with a Mars-like atmosphere (980 Pa of 95.55% CO_2_) and a total dose of cosmic ionizing radiation of about 0.5 Gy^19,36^. Mission ground reference (MGR) experiments were performed in which samples were exposed to a mean UV radiation (200–400 nm) fluence as transmitted during the space mission^35^. Since it was not possible to reproduce each different dose reaching each different position within the flight hardware^35^, MGR samples were not used for genomic comparative analysis.

### Cyanobacterial strains

*Chroococcidiopsis* sp. CCMEE 029 was isolated by Roseli Ocampo-Friedmann from cryptoendolithic communities in the Negev Desert (Israel) and maintained at the University of Rome Tor Vergata as part of the Culture Collection of Microorganisms from Extreme Environments (CCMEE) established by E. Imre Friedmann. For the BIOMEX space experiment cells of *Chroococcidiopsis* sp. CCMEE were mixed with the Martian simulant P-MRS, plated onto agarized BG-11, air-dried and integrated into the EXPOSE-R2 facility^19^. After return to Earth a 25-mm^2^ fragment of the space-exposed sample was inoculated into BG-11 medium^37^ under optimal growth conditions and the space-derived culture used for genomic DNA extraction (see below). The reference strain of *Chroococcidiopsis* sp. CCMEE 029 was grown in three independent liquid cultures (CCMEE 029 A, B and C) and used for genomic DNA extraction (see below).

### Genomic DNA extraction

Genomic DNA was extracted from three independent cultures of the ground-reference strain (CCMEE 029 A, B and C) and the space-derivate using the MOBIO PowerWater^®^ DNA Isolation Kit (MO BIO Laboratories Inc., Carlsbad, CA, USA) following manufacturer's instructions, and eluted in 25 µl of sterile bi-distilled water. The eluted DNA was quantified using the NanoDrop Lite Spectrophotometer (Thermo Scientific, Waltham, MA, USA).

### DNA sequencing and quality control

Genomic DNA was sequenced using the Illumina MiSeq and Oxford Nanopore platforms. Illumina libraries were prepared using the Kapa Hyperplus library kit (Roche Molecular Systems Inc., Pleasanton, CA, USA) following the manufacturer's instruction, the final pooled library was quantified by qPCR and sequenced on MiSeq Illumina platform using the chemical V3 PE 2 × 300. The library size of 400 bp was detected using the Agilent 2100 Tapestation. Oxford Nanopore libraries were prepared following manufacturer's instructions, samples were firstly labelled using a rapid barcoding kit SQK-RBK004 and then sequenced through ligation kit LSK-SQK109. Base calls were performed by means of the guppy software 4.4.1 (www.nanoporetech.com). Quality check was performed using FastQC^38^, version 0.11.8 and Multiqc^38,39^, version 1.8. The GC content and k-mer spectra analysis was performed through KAT suite tools^40^, version 2.4.2, to identify putative contaminants. The non-cyanobacterial sequences were identified using Kraken, an ultrafast metagenomic sequence classification^41^, and filtered out using custom Python 3 script.

### Genome assembly and gene annotation

Illumina and Nanopore reads were used together to produce an assembly using Unicycler^42^, version 0.4.8, a hybrid assembly pipeline for bacterial genomes. All the data obtained for the ground-reference triplicates were merged to improve contiguity and accuracy of the results. This assembly served throughout the present work as ground-reference genome. The same sequencing data and software were used for the assembly of the genome of the space-derived sample.

The ground-reference genome was annotated using the software PROKKA^43^, version 1.14.5, through the interface provided by Galaxy Tool^44^, version 1.14.5 (www.usegalaxy.eu). Gene function was described using terms from Gene Ontology (GO), Kyoto Encyclopedia of Genes and Genomes (KEGG) and protein domain composition from Protein Families Database (Pfam). GO terms were assigned with Blast2GO analysis^45^, version 5.2 (www.blast2go.com), while KEGG identifiers and Pfam domains were identified using BlastKOALA software^46^.

### Detection of single nucleotide variations (SNVs), insertion/deletions (InDels) and structural variants (SVs)

The Illumina reads of the ground-reference triplicates (CCMEE 029 A, B and C) and space-derivate were mapped to the ground-reference genome using the mem command of the Burrows-Wheeler Aligner (BWA) with default parameters^47^, version 0.7.17-r1188. Alignments were sorted, indexed and converted to BAM file (binary alignment map) using SAMtools, version 1.10 (www.htslib.org/), final sorted bam results were used for downstream analysis. The long reads from Oxford Nanopore for each sample (CCMEE 029 A, B, C and space-derived) were aligned against the ground-reference genome using NGMLR, version 0.2.7 (https://github.com/philres/ngmlr) with default parameters.

SNV and InDel detection was carried out by employing the multi-sample approach implemented in xAtlas^47,48^, version 0.2.1 (https://github.com/jfarek/xatlas), generating a Variant Call Format (VCF) file with the putative SNVs and InDels for each sample.

Structural Variants (SVs), bigger than a 1 kbp, were identified using three different methods. The first approach exploited the abnormal orientation of the Illumina PE reads aligned against the ground-reference genome and it was implemented in the DELLY software^49^, version 0.8.3. The second approach identified the abnormal alignment of the Oxford Nanopore Technology (ONT) reads against the ground-reference genome and was performed using Sniffles (https://github.com/fritzsedlazeck/Sniffles). The third method compared the assembled contigs and the ground-reference genome using Assemblytics^50,51^, version 1.2.1. Each software was used with default parameters.

SNVs, InDels and SVs were merged in a single VCF file that was filtered to keep only the variants with a good quality (Filter parameter in VCF file: PASS) and to exclude the variants in which all the samples shared the same genotype. All the filtering steps were performed using a custom Python script. The effects of each variant on the gene function were classified and annotated using the software SnpEff^52^, version 5.2.

### Comparative alignments

Different assemblies were compared by means of whole genome alignment using the Mummer^53^ suite for genome alignment, version 4.0 (https://github.com/mummer4/mummer).

## Supplementary Information

Supplementary Figures.

Supplementary Tables.

## Data Availability

This Whole Genome project was deposited in the NCBI BioProject, BioSample under the following accession numbers: PRJNA746498, SAMN20209045, and CP083761-CP083762. The raw sequence Nanopore and Illumina reads have been deposited in the SRA database (accession number: SRR15174960-67).
