# Supplementary material for: Absence of increased genomic variants in the cyanobacterium Chroococcidiopsis exposed to Mars-like conditions outside the space station
Source: Sci Rep. 2022 May 19;12:8437. doi: 10.1038/s41598-022-12631-5 (PMC9120168; doi:10.1038/s41598-022-12631-5)
Supplement: Supplementary file 1 — Supplementary Figures. [file 41598_2022_12631_MOESM1_ESM.doc]

**Supplementary Figures**

**Absence of increased genomic variants in the cyanobacterium *Chroococcidiopsis* exposed to Mars-like conditions outside the Space Station**

Alessandro Napoli^1‡^, Diego Micheletti^2‡^, Massimo Pindo^2^, Simone Larger^2^, Alessandro Cestaro^2^, Jean-Pierre de Vera^3^, Daniela Billi^1*^.

^1^University of Rome Tor Vergata, Department of Biology, Via della Ricerca Scientifica snc, 00133 Rome, Italy

^2^Edmund Mach Foundation via E. Mach 1, 38010, San Michele all'Adige, Italy

^3^German Aerospace Center (DLR), Microgravity User Support Center, Linder Höhe, 51147 Köln, Germany


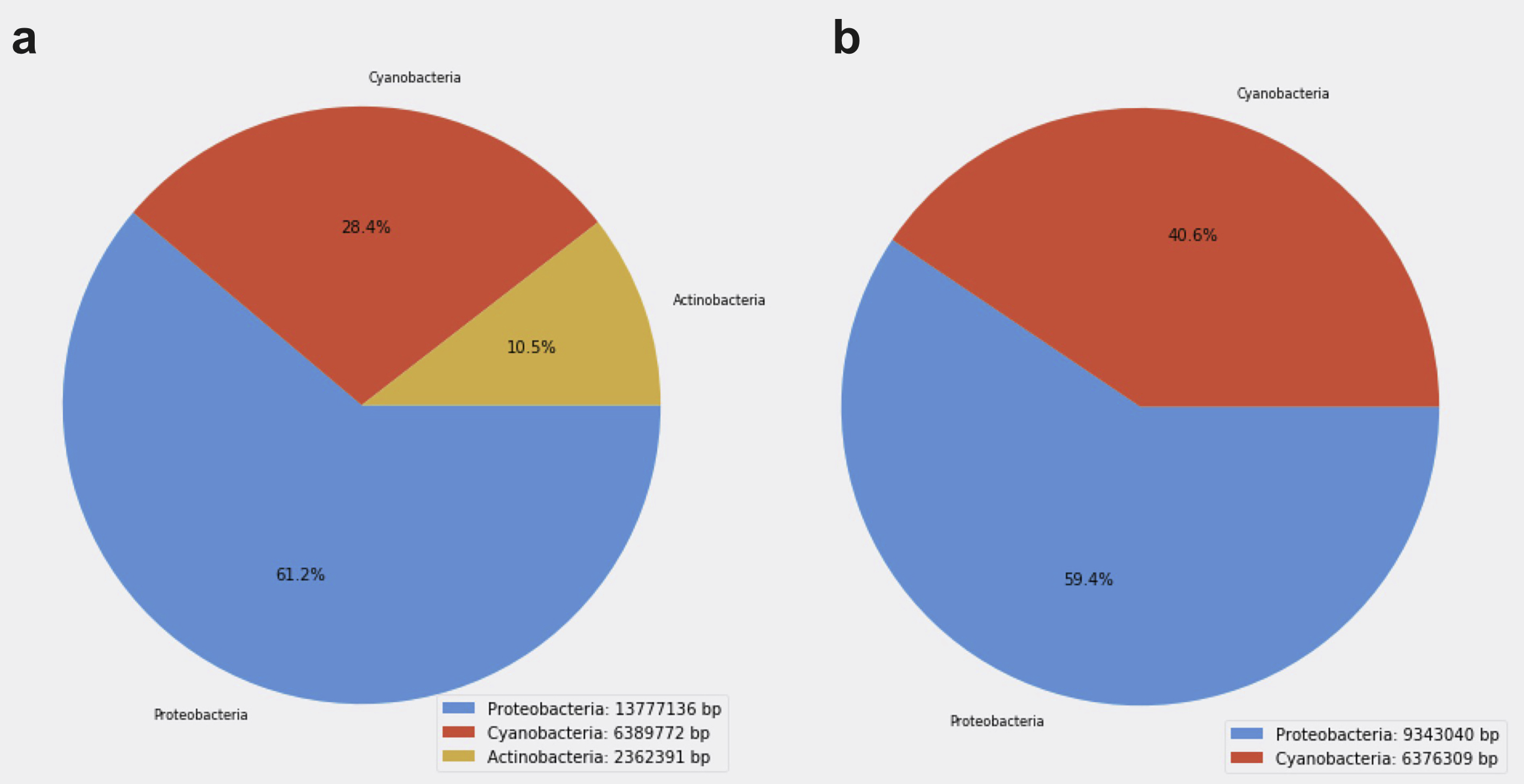


**Figure 1.** Pie Chart of size distribution of taxonomy classes in the ground-reference genome of *Chroococcidiopsis* sp. CCMEE 029 (A) and space-derivate (B).


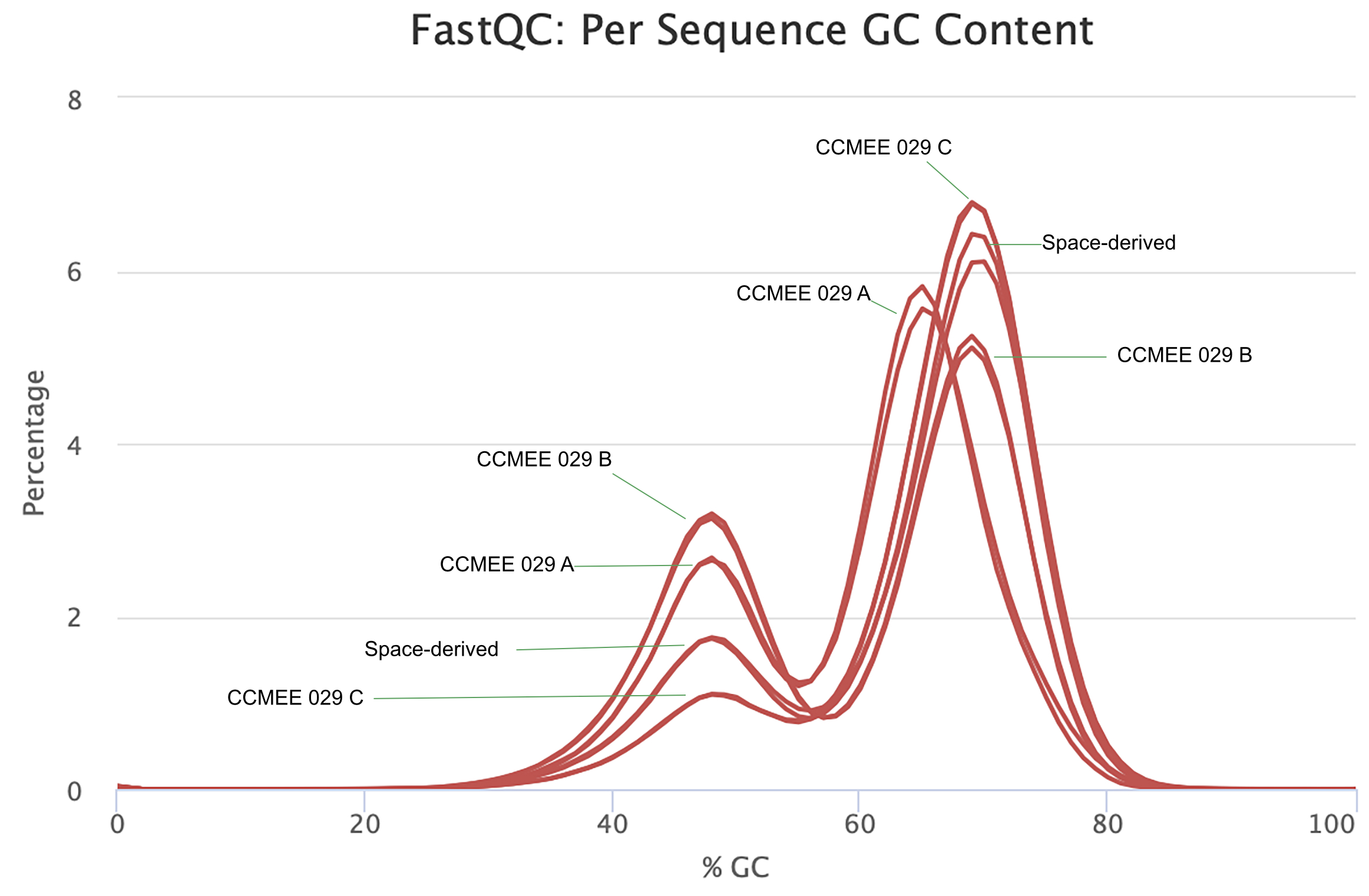


**Figure 2.** Distribution of GC content across forward and reverse reads of paired-end sequencing (Illumina MiSeq) of triplicates of the ground-reference of *Chroococcidiopsis* sp. CCMEE 029 (CCMEE 029 A, B, C) and space-derivate.


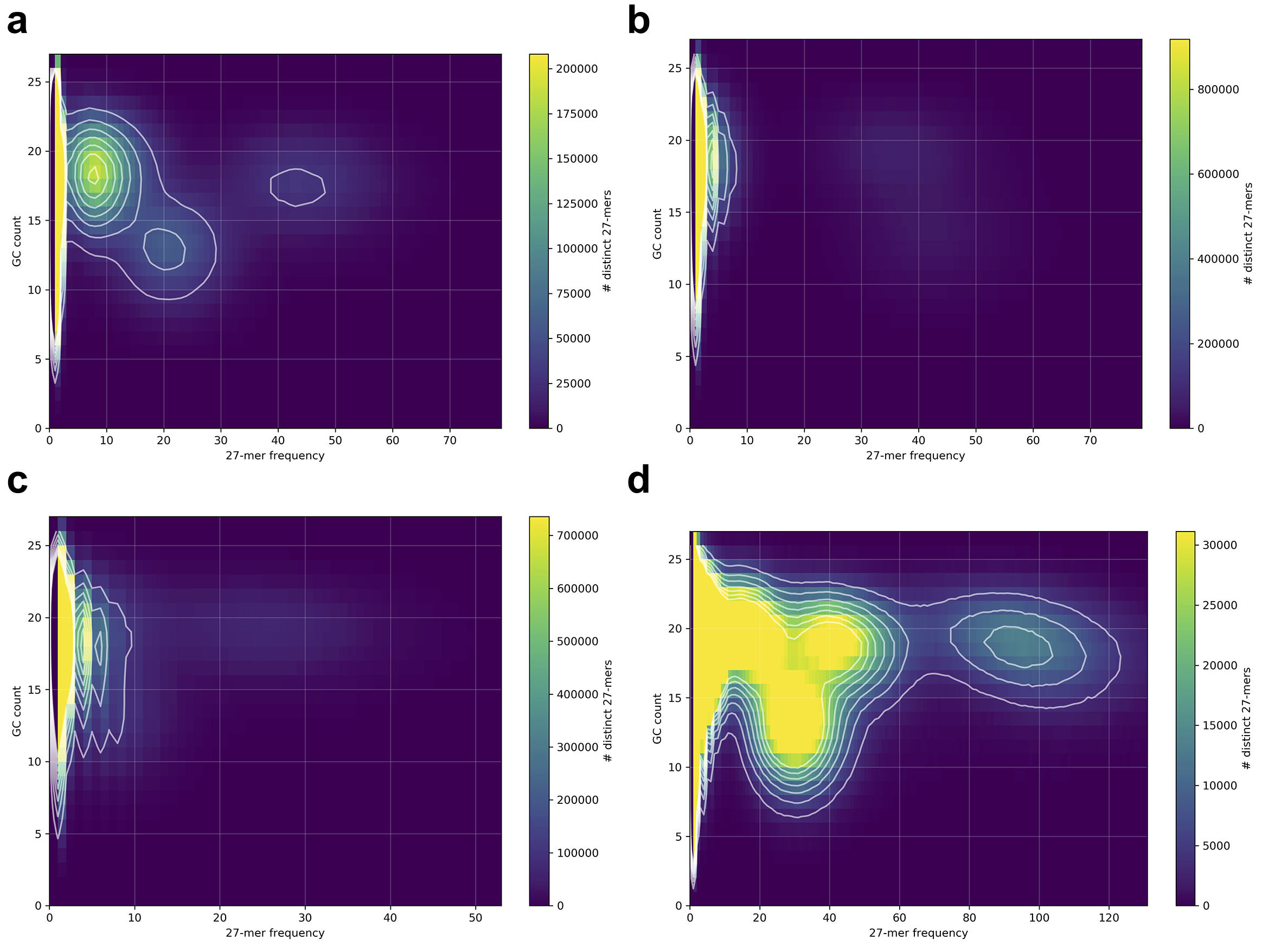


**Figure 3.** K-mer spectra analysis of the genome of triplicates of the ground-reference of *Chroococcidiopsis* sp. CCMEE 029 (A, B, C) and space-derivate (D).

**
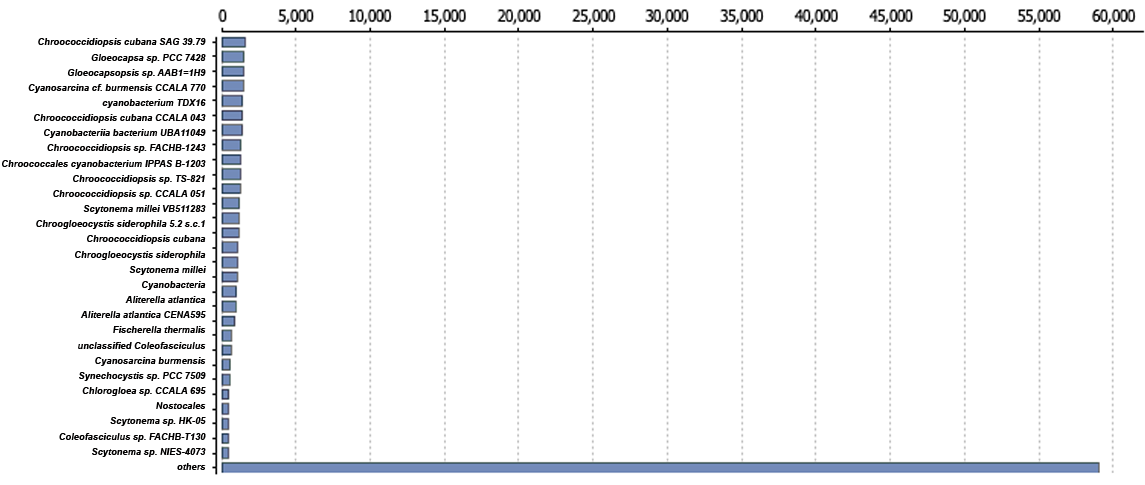
Figure 4.** Species distribution of annotated genes in the ground-reference genome of *Chroococcidiopsis* sp. CCMEE 029.


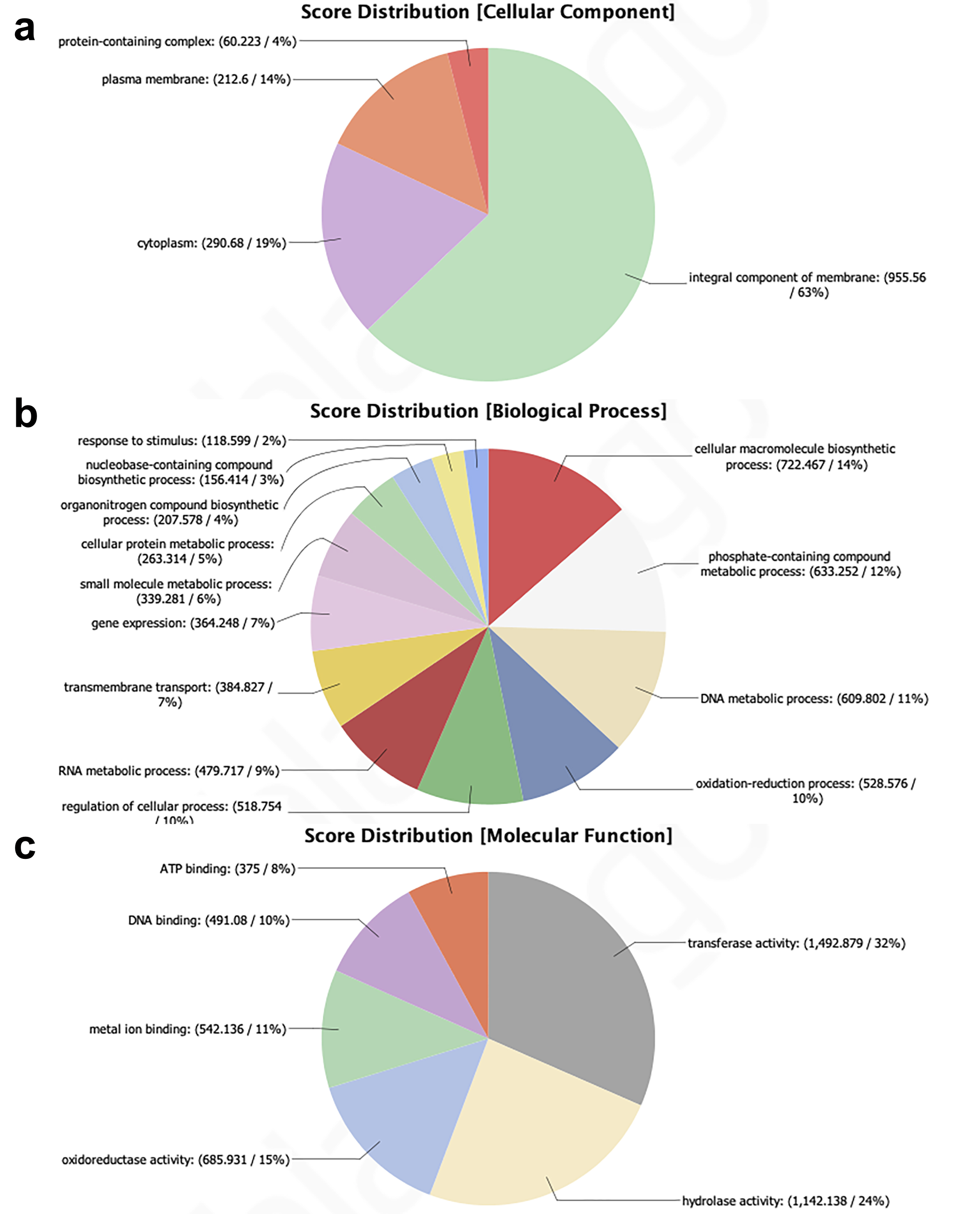
 **Figure 5.** Blast2GO annotation of the ground-reference genome of *Chroococcidiopsis* sp. CCMEE 029. Cellular component, Biological process and Molecular Function.


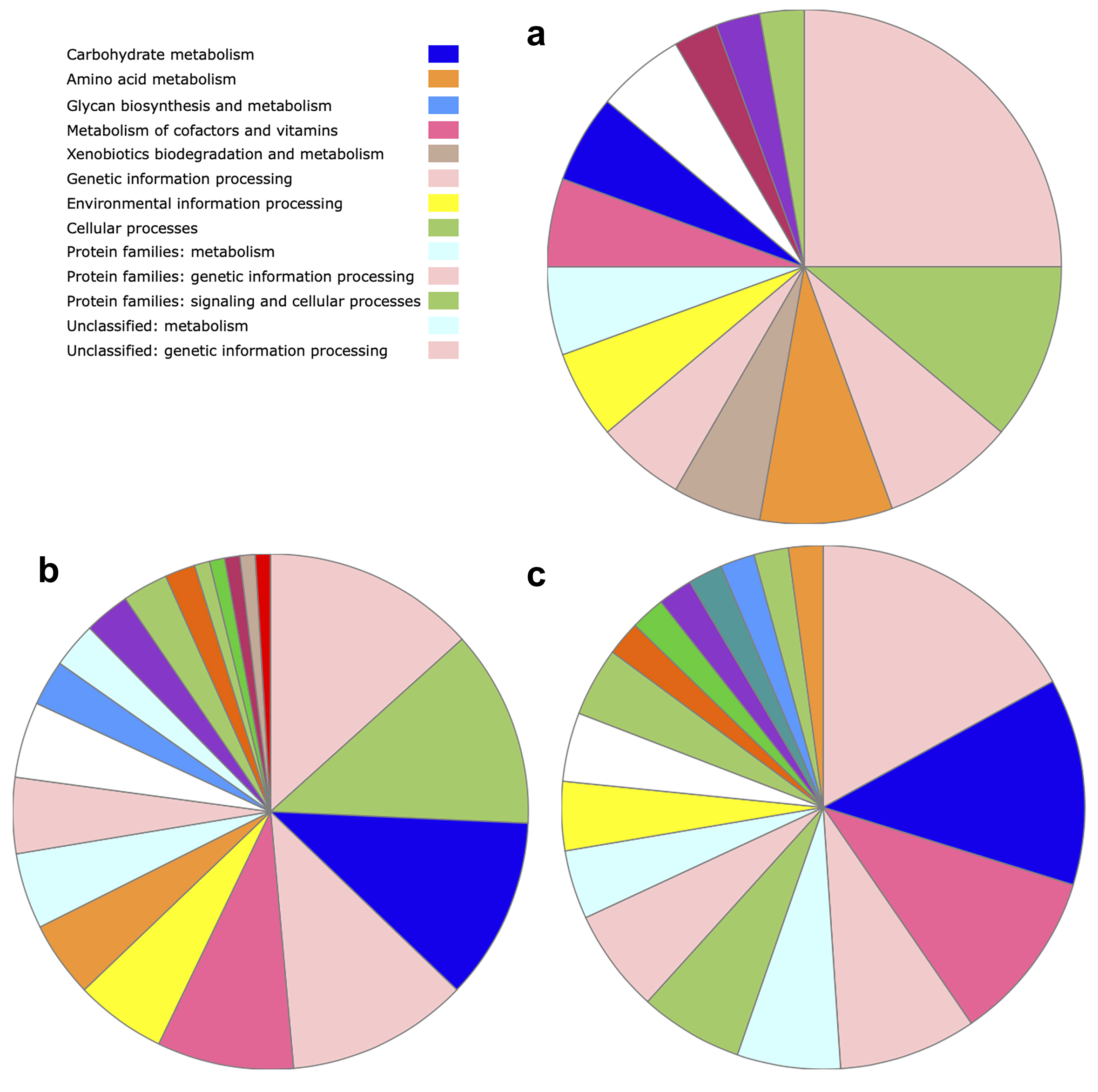
**Figure 6.** Pie Chart showing the functional categories of genes affected by variants. Affected genes shared by the ground-reference triplicates (CCMEE 029 A, B, C) and space-derivate (A.) Affected genes shared by the ground-reference triplicates (B). Affected genes in the space-derived sample.
