# Supplementary material for: Absence of increased genomic variants in the cyanobacterium Chroococcidiopsis exposed to Mars-like conditions outside the space station
Source: Sci Rep. 2022 May 19;12:8437. doi: 10.1038/s41598-022-12631-5 (PMC9120168; doi:10.1038/s41598-022-12631-5)
Supplement: Supplementary file 2 — Supplementary Tables. [file 41598_2022_12631_MOESM2_ESM.doc]

Supplementary Tables

**Absence of increased genomic variants in the cyanobacterium *Chroococcidiopsis* exposed to Mars-like conditions outside the Space Station**

Alessandro Napoli^1‡^, Diego Micheletti^2‡^, Massimo Pindo^2^, Simone Larger^2^, Alessandro Cestaro^2^, Jean-Pierre de Vera^3^, Daniela Billi^1*^.

^1^University of Rome Tor Vergata, Department of Biology, Via della Ricerca Scientifica snc, 00133 Rome, Italy

^2^Edmund Mach Foundation via E. Mach 1, 38010, San Michele all'Adige, Italy

^3^German Aerospace Center (DLR), Microgravity User Support Center, Linder Höhe, 51147 Köln, Germany

**Table 1.** Illumina and Oxford Nanopore sequencing data.

|  | **GROUND-REFERENCE** | | | **SPACE-DERIVED** |
| --- | --- | --- | --- | --- |
|  | **A** | **B** | **C** |  |
| **ILLUMINA Paired-End libraries** |  |  |  |  |
| Number of reads (*10^3) | 1.76 | 2.06 | 1.24 | 3.9 |
| Total amount of bases (bp) | 584631730 | 877878280 | 506863635 | 1276378445 |
| **Oxford Nanopore Technology** |  |  |  |  |
| Number of Reads (*10^3) | 500.4 | 96.7 | 471.8 | 135.452 |
| Total amount of bases (bp) | 2529660596 | 511009847 | 682583720 | 599374355 |
| Read length range (bp) | 116-85915 | 139-35008 | 121-69457 | 98-49084 |
| Read length median (bp) | 2000-3999 | 2000-3999 | 2000-3999 | 2000-2999 |

**Table 2.** Consensus assembly of ground-reference and space-derivate of *Chroococcidiopsis* sp. CCMEE 029. Sum Length, N50 and NG50 expressed in Kbp.

|  | **Contigs** | **Sum Legnth (Kbp)** | **N50**  **(Kbp)** | **NG50 (Kbp)** |
| --- | --- | --- | --- | --- |
| **CCMEE 029 Ground-reference assembly**  **(before filtering)** | 115 | 22552,326 | 984400 | 5716410 |
| **CCMEE 029 Space-derived assembly (before filtering)** | 75 | 15743,262 | 1002848 | 4897168 |

**Table** **3**. Kraken classification of ground-reference genome of *Chroococcidiopsis* sp. CCMEE 029. Data assembly before filtering.

| **Taxon** | **Sum Size (Kbp)** | **Number of Contigs** |
| --- | --- | --- |
| Nostoc punctiforme PCC 73102 | 5716410 | 1 |
| Pseudoxanthomonas suwonensis 11-1 | 4175584 | 9 |
| Zymomonas mobilis subsp. mobilis NCIMB 11163 | 3864452 | 1 |
| Brevundimonas subvibrioides ATCC 15264 | 3104270 | 4 |
| Clavibacter michiganensis subsp. michiganensis NCPPB 382 | 792086 | 8 |
| Ralstonia pickettii 12D | 760185 | 1 |
| Clavibacter michiganensis subsp. sepedonicus | 754510 | 12 |
| Chroococcidiopsis thermalis PCC 7203 | 673362 | 1 |
| Clavibacter michiganensis subsp. nebraskensis NCPPB 2581 | 613601 | 7 |
| Pseudomonas mendocina NK-01 | 612111 | 11 |
| Pseudomonas mendocina ymp | 451744 | 9 |
| Alicycliphilus denitrificans BC | 212088 | 2 |
| Methylobacterium populi BJ001 | 162300 | 1 |
| Sphingopyxis alaskensis RB2256 | 130113 | 1 |
| Acidovorax sp. JS42 | 104560 | 1 |
| Rhizobiales | 86729 | 1 |
| Leifsonia xyli subsp. cynodontis DSM 46306 | 85139 | 1 |
| Microbacterium testaceum StLB037 | 55600 | 2 |
| Microbacteriaceae | 29315 | 1 |
| Cellvibrio gilvus ATCC 13127 | 25131 | 1 |
| Rhodobacter capsulatus SB 1003 | 24898 | 1 |
| Stenotrophomonas maltophilia D457 | 17856 | 1 |
| Komagataeibacter medellinensis NBRC 3288 | 11677 | 1 |
| Anaeromyxobacter sp. Fw109-5 | 10972 | 1 |
| Pseudomonas sp. VLB120 | 8590 | 1 |
| Acidiphilium | 8412 | 1 |
| Bradyrhizobium japonicum USDA 6 | 5759 | 1 |
| Intrasporangium calvum DSM 43043 | 4851 | 1 |
| Rhodanobacter denitrificans | 4583 | 1 |
| Granulibacter bethesdensis CGDNIH1 | 4019 | 1 |
| Rhodopseudomonas palustris HaA2 | 3761 | 1 |
| Bradyrhizobium | 3629 | 1 |
| Leptothrix cholodnii SP-6 | 3398 | 1 |
| Hyphomicrobium denitrificans 1NES1 | 2693 | 1 |
| Pseudomonas sp. TKP | 1935 | 1 |
| Actinoplanes sp. N902-109 | 1093 | 1 |
| Streptomyces hygroscopicus subsp. jinggangensis | 1065 | 1 |
| Xanthomonas | 818 | 1 |
| Unclassified | 23027 | 22 |

**Table 4**. Kraken classification of space-derived genome of *Chroococcidiopsis* sp. CCMEE 029 Data assembly before filtering.

| Taxon | Sum Size (Kbp) | Number of Contigs |
| --- | --- | --- |
| Nostoc punctiforme PCC 73102 | 5708862 | 4 |
| Pseudoxanthomonas suwonensis 11-1 | 4147130 | 9 |
| Novosphingobium aromaticivorans DSM 12444 | 3433002 | 3 |
| Chroococcidiopsis thermalis PCC 7203 | 663434 | 3 |
| Ralstonia pickettii 12D | 544059 | 1 |
| Novosphingobium sp. PP1Y | 414185 | 1 |
| Alicycliphilus denitrificans BC | 316022 | 1 |
| Stenotrophomonas maltophilia R551-3 | 113391 | 1 |
| Ralstonia pickettii DTP0602 | 112460 | 1 |
| Methylobacterium nodulans ORS 2060 | 95635 | 1 |
| Bradyrhizobium oligotrophicum S58 | 35303 | 1 |
| Methylobacterium sp. 4-46 | 27877 | 1 |
| Magnetospirillum gryphiswaldense MSR-1 v2 | 27819 | 2 |
| Bradyrhizobium diazoefficiens USDA 110 | 24552 | 1 |
| Rhodobacter sphaeroides ATCC 17025 | 17981 | 1 |
| Stenotrophomonas maltophilia D457 | 17856 | 1 |
| Rhodanobacter denitrificans | 5148 | 1 |
| Sinorhizobium fredii USDA 257 | 3326 | 1 |
| Azospirillum lipoferum 4B | 3276 | 2 |
| Microcoleus sp. PCC 7113 | 2393 | 2 |
| Acidovorax sp. JS42 | 2012 | 1 |
| Methylobacterium extorquens DM4 | 1879 | 1 |
| Gloeocapsa sp. PCC 7428 | 1394 | 1 |
| Anabaena variabilis ATCC 29413 | 226 | 1 |
| Proteobacteria | 127 | 1 |
| Unclassified | 23913 | 32 |

**Table 5.** Annotation with PROKKA and Blast2GO of genes affected by variants shared by triplicates of the ground-reference *Chroococcidiopsis* sp. CCMEE 029 (CCMEE 029 A, B, C) and space-derivate.

| **Prokka** | **Blast2GO** | | |
| --- | --- | --- | --- |
| **Gene Name** | **Molecular Function** | **Biological Process** | **Cellular Component** |
| IS6 family transposase |  | DNA integration |  |
| response regulator transcription factor |  | phosphorelay signal transduction system |  |
| ParA family protein | GTP binding |  |  |
| CopG domain protein DNA-binding domain protein | DNA binding |  |  |
| hypothetical protein |  |  | membrane |
| CHAT domain-containing protein |  |  | integral component of membrane |
| insertion element iso-IS1n protein insB | DNA binding |  |  |
| IS110 family transposase | DNA binding |  |  |
| IS1 family transposase | | DNA binding |  |
| glutamate--tRNA ligase | | tRNA binding |  |
| cupredoxin domain-containing protein |  |  | integral component of membrane |
| IS4 family transposase | DNA binding |  |  |
| phosphate ABC transporter substrate-binding protein PstS |  | phosphate ion transmembrane transport |  |
| catalase/peroxidase HPI | catalase activity |  |  |
| glutamine amidotransferase |  | glutamine metabolic process |  |
| GNAT family N-acetyltransferase | N-acetyltransferase activity |  |  |
| LuxR family transcriptional regulator | DNA binding |  |  |
| DUF1772 domain-containing protein |  |  | integral component of membrane |
| ribonuclease III | ribonuclease III activity |  |  |
| IS701 family transposase | | DNA binding |  |
| DUF3459 domain-containing protein |  | carbohydrate metabolic process |  |
| DUF697 domain-containing protein |  |  | integral component of membrane |
| photosystem I reaction center subunit PsaK |  |  | photosystem I |
| phosphoribosylanthranilate isomerase |  | tryptophan biosynthetic process |  |
| flavohemoprotein | oxygen carrier activity |  |  |
| protogloblin ApPgb | oxygen binding |  |  |
| MBL fold metallo-hydrolase | | hydrolase activity |  |
| ATP-dependent RecD-like DNA helicase | DNA binding |  |  |
| molybdopterin oxidoreductase family protein | oxidoreductase activity |  |  |
| nucleotidyltransferase domain-containing protein | nucleotidyltransferase activity |  |  |
| diguanylate cyclase |  | signal transduction |  |
| elongation factor Tu | translation elongation factor activity |  |  |
| penicillin acylase family protein |  |  | integral component of membrane |
| amino acid adenylation domain-containing protein | catalytic activity |  |  |
| non-ribosomal peptide synthetase | isomerase activity |  |  |
| HAD-IIIC family phosphatase | N-acetyltransferase activity |  |  |
| acyl-CoA dehydrogenase family protein | butyryl-CoA dehydrogenase activity |  |  |
| type I polyketide synthase | phosphopantetheine binding |  |  |
| iron-siderophore ABC transporter substrate-binding protein |  |  | integral component of membrane |
| ABC transporter ATP-binding protein | ATP binding |  |  |
| site-specific integrase | DNA binding |  |  |
| AAA family ATPase | phosphorelay sensor kinase activity |  |  |
| DsbA family protein |  |  | protein kinase CK2 complex |
| SDR family oxidoreductase | oxidoreductase activity |  |  |
| VOC family protein | dioxygenase activity |  |  |
| nuclear transport factor 2 family protein |  | polyketide metabolic process |  |
| glucose 1-dehydrogenase | 3-oxoacyl-[acyl-carrier-protein] reductase (NADPH) activity |  |  |
| GMC family oxidoreductase N-terminal domain-containing protein | oxidoreductase activity, acting on CH-OH group of donors |  |  |
| alpha/beta hydrolase | hydrolase activity |  |  |
| NAD(P)/FAD-dependent oxidoreductase |  |  | integral component of membrane |
| cupin domain-containing protein | isomerase activity |  |  |
| type 1 glutamine amidotransferase domain-containing protein | transferase activity |  |  |
| CPBP family intramembrane metalloprotease | metalloendopeptidase activity |  |  |
| class A beta-lactamase, subclass A2 | beta-lactamase activity |  |  |
| type I restriction endonuclease subunit R | DNA binding |  |  |
| IS5/IS1182 family transposase |  |  | membrane |
| winged helix-turn-helix transcriptional regulator | DNA-binding transcription factor activity |  |  |
| DoxX family membrane protein |  |  | membrane |
| xylulokinase | xylulokinase activity |  |  |
| hydrolase | hydrolase activity |  |  |
| NAD-dependent epimerase/dehydratase family protein | L-threonine 3-dehydrogenase activity |  |  |

**Table 6.** Annotation of genes with stop codons gained in triplicates of the ground-reference *Chroococcidiopsis* sp. CCMEE 029 (CCMEE 029-A, B, C) and space-derivate.

|  | **Ground-reference** | | | **Space-derivate** |
| --- | --- | --- | --- | --- |
| **KEGG** | **A** | **B** | **C** |  |
| IS6 family transposase | 2 | 2 | 2 | 2 |
| VOC family protein | 1 | 1 | 0 | 1 |
| GNAT family N-acetyltransferase | 1 | 1 | 1 | 1 |
| sulfate ABC transporter substrate-binding protein | 1 | 0 | 0 | 0 |
| IS701 family transposase | 1 | 1 | 0 | 0 |
| RidA family protein | 1 | 1 | 1 | 0 |
| SRPBCC domain-containing protein | 1 | 1 | 1 | 0 |
| M24 family metallopeptidase | 1 | 0 | 0 | 0 |
| phosphoenolpyruvate synthase | 1 | 0 | 0 | 0 |
| DUF3352 domain-containing protein | 1 | 0 | 0 | 0 |
| hypothetical protein | 1 | 0 | 0 | 0 |
| Unidentified | 1 | 1 | 0 | 1 |
| IS1 family transposase | 1 | 1 | 0 | 1 |
| IS630 family transposase | 0 | 0 | 0 | 1 |
| WecB/TagA/CpsF family glycosyltransferase | 0 | 0 | 0 | 1 |
